# Tissue-resident lymphocytes: weaponized sentinels at barrier surfaces

**DOI:** 10.12688/f1000research.25234.1

**Published:** 2020-07-09

**Authors:** Gabrielle T. Belz, Renae Denman, Cyril Seillet, Nicolas Jacquelot

**Affiliations:** 1 The University of Queensland, Diamantina Institute, Brisbane, Queensland, 4102, Australia; 2 Walter and Eliza Hall Institute of Medical Research, Parkville, Melbourne, Victoria, 3052, Australia; 3 Department of Medical Biology, The University of Melbourne, Melbourne, Victoria, 3010, Australia

**Keywords:** memory T cells, tissue-resident cells, immune protection, barrier protection, pathogens

## Abstract

Tissue-resident immune cells stably localize in tissues largely independent of the circulatory system. While initial studies have focused on the recognition of CD8 ^+^ tissue-resident memory T (CD8 T _RM_) cells, it is now clear that numerous cell types such as CD4 ^+^ T cells, gd T cells, innate lymphoid cells and mucosal-associated invariant T (MAIT) cells form stable populations in tissues. They are enriched at the barrier surfaces and within non-lymphoid compartments. They provide an extensive immune network capable of sensing local perturbations of the body’s homeostasis. This positioning enables immune cells to positively influence immune protection against infection and cancer but paradoxically also augment autoimmunity, allergy and chronic inflammatory diseases. Here, we highlight the recent studies across multiple lymphoid immune cell types that have emerged on this research topic and extend our understanding of this important cellular network. In addition, we highlight the areas that remain gaps in our knowledge of the regulation of these cells and how a deeper understanding may result in new ways to ‘target’ these cells to influence disease outcome and treatments.

## Introduction

The immune system is composed of millions of diverse cell types distributed amongst the circulatory systems and the tissues. Immune cells found in the blood and lymphoid tissues are the primary anatomic compartments that have been studied and form the foundation of our understanding of immune cell homeostasis. Indeed, recirculation of memory T cells is a hallmark of their functional capacity to affect their protective roles during immunosurveillance. Despite this, the vast majority of immune cells are not located in the circulation trafficking between the lymphoid tissues and blood or lymph. Instead, they are localized in non-lymphoid tissues where they can reside for extensive periods of time. The significant concentration of immune cells within the barrier tissues—the skin and digestive, reproductive and respiratory tracts—pivotally positions antigen-experienced cells to mediate local responses against antigenic and pathogenic challenges. The identification of ‘tissue-resident’ T cells resolved a major problem in the immune system by positioning ‘primed’ cytolytic cells in tissue compartments where they would first encounter a pathogen or breach the body’s surface.

But is tissue residency limited to CD8 ^+^ T cells? Despite the attraction of such a thesis, there is little evidence that this type of programing should be restricted to a single-cell subset when the immune system is colonized by a myriad of diverse cells, many of which occur at mucosal or barrier surfaces. If the program is broader, how is it regulated and is it fixed such that immune cells are predestined to a tissue-resident fate? Or is there plasticity in the system enabling resident cells to become mobile, and for mobile cells to change tactics and become sedentary?

Multiple cell types are now recognized to be highly enriched at the body’s barrier surfaces and in non-lymphoid tissues. This includes classic adaptive thymic-derived conventional and regulatory CD4 ^+^ and CD8 ^+^ T-cell subsets together with different subsets of innate immune cells and so-called non-conventional lymphocytes such as γδ T cells, natural killer (NK) T cells, mucosal-associated invariant T (MAIT) cells and CD8αα intra-epithelial lymphocytes. Our understanding of these different cell types is beyond the scope of this review but they provide important clues in understanding immune system recognition of antigenic types and how border protection might have evolved evolutionarily. Indeed, the emergence of the discoveries and functions around these diverse cell types opens the door to unexpected targeting approaches to temporally regulate immune cell subtypes and provide new strategies for harnessing control of infectious agents and elimination of tumor cells.

Recent studies have uncovered a number of the mechanisms that regulate the temporal positioning of tissue-resident cells, but they have also revealed unexpected cues such as sensory detection and stromal cell signals which set the threshold for the transition of tissue-resident cell retention within local tissues where they are focused on local responses and remodelling to systemic responses affecting distant organs.

## CD8 ^+^ T-cell tissue residency: rethinking immune cell lifestyles

**Figure 1.**
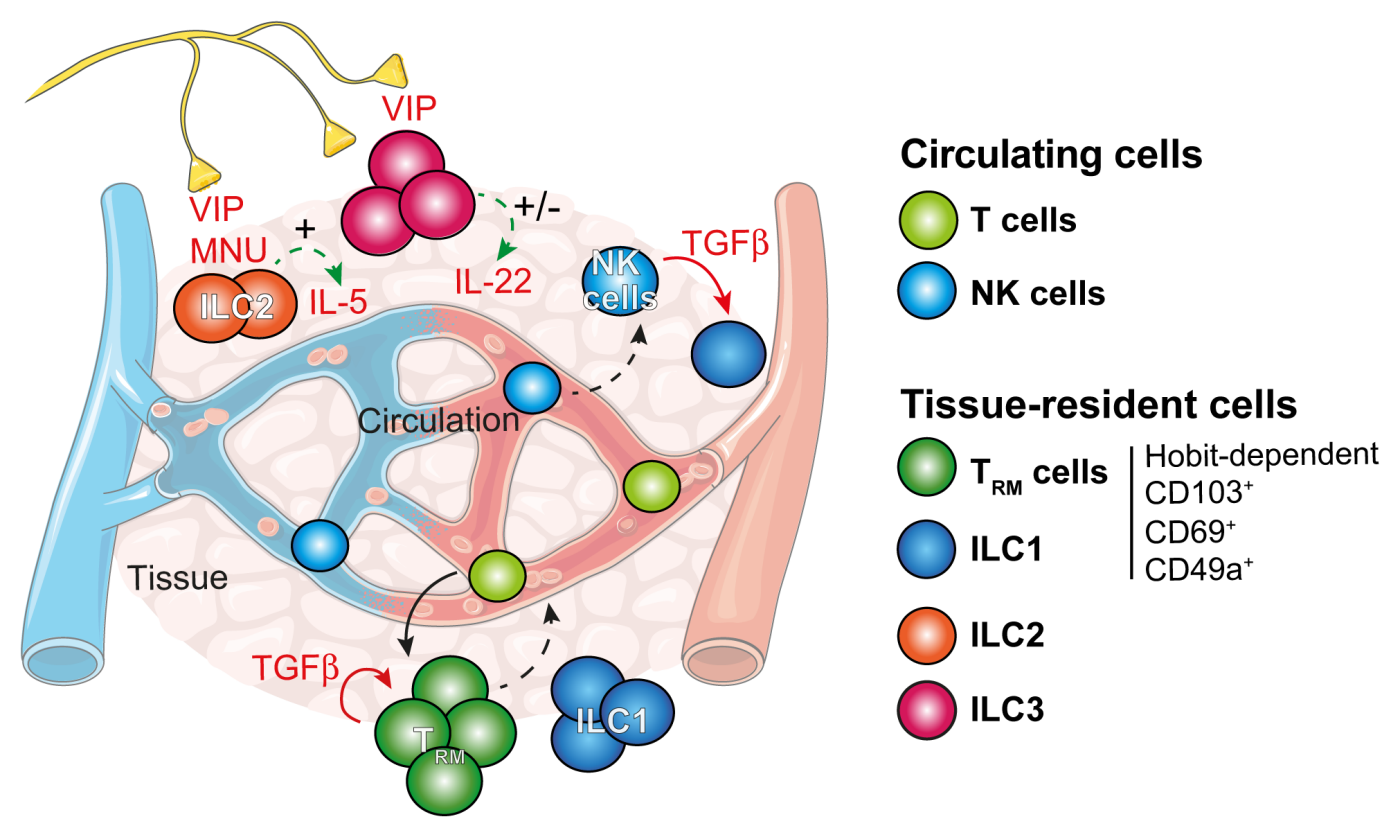
Tissue residency and modulation across lymphoid subsets and tissues. Effector T cells enter peripheral and non-lymphoid tissues and establish themselves as non-recirculating tissue-resident memory T (T _RM_) cells. Transforming growth factor-beta (TGF-β) and Hobit act as master regulators of tissue residency and regulate the expression of CD103, CD69 and CD49a of tissue-resident cells such as innate lymphoid cell 1 (ILC1) and T _RM_ cells. Natural killer (NK) cells appear to continuously circulate around the body. In the presence of TGF-β, NK cells can transdifferentiate into ILC1-like cells. ILC1, 2 and 3 are generally non-circulating and establish in tissues. Their activity is intimately modulated by numerous tissue-related factors. These include neuropeptides such as vasoactive intestinal peptide (VIP) and neuromedin U (NMU) that can activate (+) or inhibit (−) cytokine secretion. In response to an immunological threat, T cells and ILCs can relatively readily become mobile and exit tissues via the lymphatics to join circulating immune cells. Nevertheless, these cells exhibit a preference for returning to their ‘tissue of origin’ when they re-establish tissue residency. IL, interleukin.

The initial description of ‘tissue-resident’ T cells was based on the identification of specific markers that were deemed to reflect stable positioning in tissues (CD103, CD49a and CD69) and the lack of molecules associated with tissue egress and migration to secondary lymphoid organs (Klf2, S1Pr1, CCR7 and CD62L) ( Figure 1). The localization of tissue-resident memory cells within tissues, particularly at barrier surfaces, theoretically positions them to be able to initiate a faster immune response towards a pathogen without the necessity to engage other immune or stromal cells ^1^. The notion is that resident cells can undergo extensive proliferation within the tissues, allowing them to replenish, but that they do not appear to accumulate, remaining numerically stable over time ^2– 4^. This concept is supported by the failure to detect significant movement of cells within a tissue in stark contrast to effector memory T cells that are found largely in the blood and patrol the body ^2, 5– 7^. Recent evidence indicates that tissue preparation approaches greatly influence the detection of ‘tissue-resident’ cells, significantly underestimating their prevalence ^8^. Furthermore, in peripheral sites, many T cells did not express CD103 or CD69, indicating that these molecules are not universal markers and their expression reflects site-specific characteristics ^8^. It also highlights that these other T-cell populations exist in these sites and are likely to play an important role in rapid responses to secondary challenges. T cell analyses based on CX3CR1 expression revealed that effector memory (T _EM_) cells (CX3CR1 ^+^) themselves are largely excluded from tissues while central memory (T _CM_) cells and CX3CR1 ^intermediate^ cells homed to lymph nodes but that CX3CR1 ^intermediate^ cells were the dominant cell surveying peripheral tissues ^9^. Furthermore, central memory cells are enabled to migrate to non-lymphoid tissues and form the predominant population in these tissues following inflammation ^10^. This capability results from induction of the expression of E- and P-selectins due to interleukin (IL)-15-stimulated enzymatic synthesis of core 2 O-glycans that regulate CD8 ^+^ T-cell migratory behaviour ^10^. These studies highlight that tissue residency is not a static state for T cells or even other lymphocyte subsets.

Tissue-resident memory T (T _RM_) cells within peripheral tissues can not only mobilize to adjacent tissues but can also re-join the circulating memory T cell masses to maintain a stable equilibrium. Indeed, Fonseca *et al.* ^11^ recently examined this in detail and demonstrated that small intestinal tissue-resident memory cells could re-enter the circulating pool of cells and exhibited the capacity to differentiate into effector and central memory cells. Although these memory cells contribute to the overall pool of T cells, they exhibit a predilection for homing back to the tissue of origin following reactivation consistent with very early studies in the field that showed a similar phenomenon in which distinct immune cell subsets display restricted and often tissue-selective patterns of recirculation ^12^. Thus, tissue-resident lymphocytes are distinctive by their location in non-lymphoid tissues but do appear able to undergo some recirculation through a tightly regulated chemokine receptor and integrin expression pattern.

In this recent work examining the distribution of T cells in mice ^11^ and NK cells in humans ^13^, tissue-resident cells exhibited a less activated phenotype compared with their more centrally deployed counterparts and this has led to the notion of ‘outside-in’ activation ^11^. Studying the behaviour of immune cells in the face of the onslaught by an invading pathogen trying to kill us brings our attention into sharp focus on the armoury of effector molecules used to mitigate a pathogen invasion. This, it would seem, is a rather artificial situation, however, as it reflects a last-ditch effort by the innate and adaptive immune system to fight back. It is much more greatly appreciated now, though, that disease is a relatively rare state—an organism’s health depends on the maintenance of day-to-day homeostasis. Accompanying this is the very tight regulation of effector responses in tissue-resident cells and more subtle expression profiles of a number of molecules ^11, 13^. The checks-and-balances by molecular regulators within the tissues, immune cells and non-immune cells, such as neurons, physiologically integrate signals that implement activation and simultaneously restrict the development of immunopathology. To date, our understanding of these tissue signals are not well characterised, and they are of significant interest in understanding how barrier immune health is maintained.

## Lung-resident memory T cells

Lung-resident memory T cells are important to delay the spread of pathogens and recruiting recirculating T _EM_ cells. They are known to play a vital role in mounting protection against respiratory viral, bacterial and parasitic infections, including influenza virus ^14^, coronaviruses such as SARS-CoV and MERS-CoV ^15, 16^, *Bordetella pertussis* ^17^ and *Nippostrongylus brasiliensis* ^18, 19^. T _RM_ cells expand during infection and specifically target infected cells ^17^. In some cases, this response eliminates the virus and drives resolution of the illness but, in the case of influenza and coronaviruses, can lead to highly vigorous responses that induce severe pathology and may be lethal. In the lungs, T _RM_ cell populations include CD8 ^+^ T _RM_, CD4 ^+^ T _RM_ and regulatory T (T _reg_) cells. CD8 ^+^ T _RM_ cells are found in the epithelial layer of the airways and the lung parenchyma involved in gas exchange ^1, 20^. However, the main cell type that affords protection in the lung parenchyma is the CD4 ^+^ T _RM_ cell both following lung infection and in providing protection in vaccine models ^1, 14, 15, 17, 19, 21– 23^. CD8 ^+^ T _RM_ cells have also been identified as a useful target for vaccines. CD8 ^+^ T _RM_ cells located in the lung parenchyma are a more appropriate target for vaccine strategies as epithelial T _RM_ cells are short-lived and the capacity to develop new niches for this cell population is important prior to vaccination ^20, 21, 24, 25^. Following lung injury, CD8 ^+^ T _RM_ cells have been found to localize in niches where tissue regeneration has occurred and have been referred to as repair-associated memory depots ^20^. CD4 ^+^ T _RM_ cells, however, localize to the airways or around B-cell follicles and form clusters in inducible bronchus-associated lymphoid tissue (iBALT) where they can affect long-term immune protection ^20, 26^.

Lung CD4 ^+^ T _RM_ cells have been shown to be maintained at constant numbers over time following allergen exposure ^26, 27^. They have also been found to expand in the lungs during *B. pertussis* infection and more rapidly after reinfection ^17, 28^. In contrast, CD8 ^+^ T _RM_ cell populations appear to wane over time ^24, 29^. Thus, CD8 ^+^ T _RM_ cells in the epithelium of the airways must be replenished from recirculating T _EM_ cells ^29^ or from CD8 ^+^ T _RM_ cells in the lung parenchyma ^20^. This is likely due to a process in the lungs where tissue-resident cells in the epithelium are continuously cleared by phagocytic cells or via mucociliary clearance ^29^. In the case of respiratory infections such as influenza and respiratory syncytial virus, this might explain in part why complete protection is not afforded in individuals with secondary infection ^24^. T _reg_ cells in the lungs have been found to permanently reside in tissues. This has been confirmed by their expression of CD69 and CD103, which are markers of tissue residency ^30^. CD103 (αE), an integrin protein encoded by the gene *Itgae*, is not simply a marker of tissue residency but can heterodimerize with the integrin beta 7 (β7) to form the molecule αEβ7. This complex confers specificity for binding to E-cadherin and thus acts as a tether for cells in the epithelium, including CD8 ^+^ T _RM_ cells ^31^. Resident T _reg_ cells have been found to protect against lung injury and evidence has shown that they communicate with other tissue-resident cell types that together promote lung homeostasis ^30^. T _reg_ cells are located in iBALT and function by inhibiting B-cell responses, which can be beneficial in cases such as lung transplantation, where they prevent alloimmune responses ^32^. Therefore, lung T _RM_ cells are an important cell type that could be used as a future target for vaccines for respiratory infections.

## Transcriptional regulation in tissue-resident T cells

Tissue-resident cells exhibit a number of features that are distinct from their circulating counterparts. The hallmark molecular profile of tissue-resident cells is a shared expression of genes encoding adhesion ( *Itga1* and *Itgae*) and immunoregulatory ( *Cd244*, *Icos* and *Ctla-4*) molecules together with downregulation of genes required for tissue egress, such as *S1pr1* (which encodes the receptor S1P1 for sphingosine 1-phosphate), which is regulated by Krüppel-like factor 2 ^33^. Indeed, enforced expression of S1PR1 in CD8 ^+^ T cells results in a phenotype that no longer reflects tissue-resident cells ^33^. Similarly, downregulation of Eomes (encoded by *Eomesodermin*) and T-bet (encoded by *Tbx21*) expression through transforming growth factor-beta (TGF-β) responsiveness appears necessary to maintain tissue residency ^34^. This suggests that repression of S1PR1 is essential to the tissue-resident phenotype but also implies that the program is not fixed. Other transcription factors such as Hobit (homolog of B lymphocyte-induced maturation protein, Blimp-1) in T cells (Hobit; also known as *ZFP683*) are not individually required for tissue-resident cells in tissues, but loss of Hobit combined with Blimp-1 (encoded by *Prdm1*), which alone is normally associated with terminal differentiation of CD8 ^+^ T cells, revealed a more centrally regulated program required by some populations such as tissue-resident cells ^35^, whereas lung-resident cells depended more strongly on Blimp-1 ^36^. Precisely how these factors are all regulated is still unclear, particularly in the case of Hobit as expression patterns in humans appear quite different from those in mice ^37, 38^. Indeed, detailed analyses of the transcriptome of different T-cell populations that had already encountered antigens demonstrated that the central memory and tissue-resident memory cells exhibited a highly similar epigenetic program and were distinct from recently activated effector cells. This program indicated that cells exhibit considerable plasticity enabling tissue-resident cells to re-join the circulatory pool of CD8 ^+^ T cells although they were heritably imprinted to favour homing to their originating tissues ^11^.

Most studies that have examined the developmental profile (effector and memory fate decisions) and localization of lymphocytes have been predicated on the notion that the starting point is a relatively homogeneous population for which fate outcomes are defined by stochastic and environmental or external triggers. Although this approach simplifies modelling outcomes, several studies suggest that heterogeneity may reflect the several waves of layering that occur during the developmental distribution of activated fetal-derived cells across different tissues ^39– 42^. However, underlying intrinsic programs appear to be already established in fetal cells. Elegant analyses of ‘time stamped’ fetal and adult CD8 ^+^ T cells highlight this early establishment of diversity which establishes the blueprint from which subsequent tissue-specific shaping in response to environmental and pathogen challenge occurs ^41^. These temporal fate-mapping approaches combined with extensive single-cell multi-tissue transcriptional analyses in T cells ^41^, innate lymphoid cells (ILCs) ^39, 43^ and monocytes ^40^ comparing fetal and adult lymphocytes have been critical in uncovering this additional level of complexity. Unfortunately, we do not generally have markers with good resolution to distinguish fetal from adult cells and this will be necessary to comprehensively integrate the many layers of programming that contribute to the effector function and localization properties of protective immune cells.

## Tissue residency: not just for adaptive cells

Although the first discovery of tissue residency was uncovered in CD8 ^+^ T cells ^6, 44– 47^, non-adaptive immune cells also populate mucosal and peripheral sites following antigen encounter, suggesting that localization of innate cells is necessary for optimal protective immunity to pathogens. NK cells have classically been considered to circulate through the body, allowing them to patrol tissues and localize and destroy transformed or virally infected cells. However, a very recent global survey of different anatomical sites for human NK cell subsets revealed that these cells exhibit tissue-specific phenotypes and distributions that varied across age, sex and exposure to infection such as cytomegalovirus ^13^. Thus, NK cells, like T cells, can also be positioned at the front line for pathogen encounters and the features of these cells are extensively and specifically shaped by their tissue localization. Molecularly, they exhibited transcriptional signatures that included the genes *CCR7*, *SELL*, *CXCR3* and *CCR5* providing post codes for tissue-specific localization and *TCF1* and *LEF1* enabling them to maintain populations at the tissue site through homeostatic proliferation. Thus, in addition to expressing many effector molecules that align NK cells with CD8 ^+^ T cell function, they have a similar distribution in the body.

## Tissue-resident but not immobile

The term ‘tissue residency’ implies that cells are not mobile. It reflects that cells remain generally confined within a single tissue. However, it is clear that a cell’s existence in a tissue is far from static. Conventional NK cells are highly mobile. Other subsets of ILCs or their precursors, however, are distributed to the tissues during the perinatal period where they undergo proliferation and appear to establish in long-term tissue-specific niches, features reflected in their transcriptome ^39^ ( Figure 1). Seeding of these tissues depends on a number of receptors, including α4β7 integrin, CXCR5, CXCR6 and (to a lesser extent) CCR7 ^43, 48– 51^. Retention within the tissues themselves is less well understood but is likely to depend on receptors similar to those tethering T cells in tissues such as CD69, which antagonizes the receptors S1PR1 ^52^, CD49a ^53^ and CD103 (αE integrin) ^54, 55^. CD49 expression by T _RM_ cells is indicative of poised cytotoxic function, but CD49a ^−^CD8 ^+^ T cells have also been identified in healthy human skin and enriched in psoriasis. This latter population is associated with IL-17 production, highlighting the dichotomy in T _RM_ cell function and receptor expression in different settings ^53^. Following from a number of studies, however, was whether ILCs undergo recirculation. Initial studies examining movement of ILCs in parabiont mice and stem cell transplantation models supported the notion that ILCs were mandatorily tissue-resident. Emerging evidence strongly argues otherwise, and although ILCs do not undergo mass migration at steady state, they do indeed respond to various stimuli and rewire their molecular programs to undergo migration ^56, 57^. It has been demonstrated that ILC2s particularly are capable of intra-tissue mobility, a critical feature that dictates effective immune responses. Mature ILC2s residing in the gut have been shown to undergo proliferation, lymph node migration and dissemination into the blood in response to activation of alarmins, such as those found during *N. brasiliensis* infection. Migration to diverse tissue sites depends on S1P-mediated chemotaxis, which is also important for NK cells ^58– 60^. Thus, local perturbations allow extrusion of ILCs for distribution to distant tissue sites ^61^. This is in addition to the capacity for ILC2s to exit the bone marrow to replenish tissue pools following IL-33 ^62^ or fungal aeroallergen challenge ^63^. Similarly, ILC3s exhibit a constant influx and egress from the cryptopatch during inflammation, a circuitry driven by stromal cell oxysterol activation of the GPR183 receptor ^64– 66^. Collectively, these studies highlight that a highly orchestrated receptor expression pattern guides the recruitment and strategic positioning of innate cells in tissues and their deployment to distant sites when local perturbations occur.

## Sensory neurons broadcast local tissue perturbations

Recent major studies have highlighted the link between the immune and nervous systems at the mucosal barrier. Both systems can sense cytokines and neurotransmitters, allowing direct communication. For example, receptors for IL-6, TGF-β, IL-1-β and tumor necrosis factor-alpha (TNF-α) are found in both the brain and enteric neurons ^67– 69^ while immune cells express receptors for neuropeptides ( Figure 1). Several neuropeptides have been described to regulate immune cell activity and maintain tissue homeostasis but also the optimal immune response during infections. ILC2s are important to initiate responses to parasitic infections and allergic reactions that are directly regulated by neuromedin U (NMU) ^70– 72^. NMUR1 is specifically expressed on ILC2s and is found in close contact with NMU-expressing cholinergic neurons ^70– 72^. NMU is induced during parasitic helminth *N. brasiliensis* infection and directly induces the production of IL-5 and IL-13 by ILC2s. In the absence of NMUR signalling, these type 2 responses are impaired, resulting in poor control of worm infection.

ILC2s are also sensitive to the vasoactive intestinal peptide (VIP), a neurotransmitter expressed in neurons found in the lung and gut ^73^. VIP can stimulate the secretion of IL-5 by lung ILC2s which regulate systemic eosinophil numbers ^74^. In turn, lung nociceptors can sense IL-5 released to promote the production of VIP. This inflammatory signalling loop needs to be tightly controlled as dysregulation can lead to the development of allergic inflammation ^75^. VIP also regulates the activity of enteric ILC3s and their secretion of IL-22 ^76, 77^. It variously upregulates ^76^ or inhibits ^77^ IL-22 production by ILC3s, depending on the study. What is clear, however, is that VIP is induced after ingestion of food and this directly links information from the digestive system to enteric ILC3s driving their function ^76, 77^. Dampening the VIP signal to ILC3s increases susceptibility to inflammation-induced gut injuries and infection ^76, 77^. This rapid delivery of information is affected by clustering of ILC3s around VIP ^+^ neurons providing a mechanism to rapidly influence ILC3 activity ^76^.

While the nervous system can sense pathogens to activate immune cells in tissues, it also provides negative feedback loops which act to protect the host by preventing excessive inflammation that could lead to chronic inflammation. For example, beta-adrenergic receptors are activated by norepinephrine that inhibits ILC2 proliferation and function ^78^. This mechanism may function as a molecular rheostat to fine-tune the ILC2 response. Vagal nerve activation can modulate the secretion of pro-inflammatory cytokines in macrophages ^79^. This in turn leads to a negative regulatory loop which controls inflammation through the release of acetylcholine. Vagal disruption induces a reduction in the number of ILC3s in the peritoneum and in the protectin biosynthetic pathway, PCTR1 ^80^. PCTR1 is a pro-resolving mediator produced by ILC3s in response to acetylcholine and disruption of this pathway delays the resolution of inflammation associated with infection ^80^.

Collectively, these studies highlight the critical crosstalk that occurs between the immune and nervous systems which is necessary to both initiate and control the immune activity during inflammation. Disruption in this crosstalk leads to the development of chronic inflammation or suboptimal immune responses to pathogen infections ^73– 78, 80^.

## Resident innate cells are new potent drivers of tumor protection and immune targeting

Tumor formation results from a lack of detection and/or eradication of transformed cells by immune cells, leading to the progressive emergence of malignant tumors ^81, 82^. A combination of host- and tumor-related mechanisms is responsible for the development of neoplasms ^83^. These seminal studies that have built our current view of modern tumor immunology have markedly increased our understanding of tumor immunity, but the underlying models were built on the study of systemic immunity largely neglecting more localized immune cell contributions, particularly those driving early peripheral immunosurveillance. Recent investigations using high-throughput cellular and molecular methods have shed light on the enormous diversity of immune cell types within tissues ^84, 85^, including tumors ^86– 88^. Critically, this has provided insights into the role of tissue-resident cells demonstrating that tumor-infiltrating CD103-expressing T cells that align to a tissue-resident memory cell phenotype could be found in multiple cancer types ^89– 93^. While CD69 and CD103 are commonly used to identify T _RM_ cells, these markers might also be upregulated on activated tumor-infiltrating T-cell subsets in the context of tumors. Thus, it remains unclear whether CD69 ^+^CD103 ^+^T cells are true T _RM_ cells or alternately the expression of these molecules identifies effector T-cell subsets that have infiltrated the tumor bed where abundant TGF-β found in a large quantity in many tumors drives CD103 expression. Nevertheless, numerous studies have found a positive association between tumor CD69 ^+^CD103 ^+^T-cell infiltration and clinical outcomes, suggesting the beneficial role of this immune cell population in restricting tumor development and therapeutic responses ^86, 88, 94– 98^. In addition, tumor-infiltrating T _RM_ cells express several immune checkpoint molecules (for example, CTLA-4, TIGIT, TIM-3, LAG3 and programmed cell death-1 [PD-1]), indicating that they might respond effectively to immune checkpoint blockers ^86, 88, 98^. This is highlighted by the association of tumor enrichment of T _RM_ cells, or in genes preferentially expressed by T _RM_ cells, during anti-PD-1 therapy with increased responsiveness to treatment ^88, 94, 99^. However, despite the expression of immune checkpoint molecules, tumor T _RM_ cells do not seem to harbor an exhausted phenotype and express high levels of IL-2, interferon gamma, TNF-α and cytotoxic molecules ^99^. These cells also proliferated more and had reduced T cell receptor diversity compared with non-T _RM_ cells. In addition, increased clonal expansion was observed which might be associated with the specific recognition of tumor-associated antigens that would drive antigen-specific T _RM_ cell proliferation ^99^. However, formal identification of antigen-specific T _RM_ cells through tetramer staining is required to confirm that these cells are not exhausted and are able to secrete a large amount of pro-inflammatory cytokines and cytotoxic molecules upon antigen re-exposure. Collectively, T _RM_ cells represent an emerging and highly valuable immune cell population with potent effector functions important in anti-tumor immunity.

In addition to identifying adaptive resident T lymphocytes, pioneering work has identified tissue-resident ILCs in both murine and human tumors ^100– 103^. Our understanding of the characteristics of these cells in the cancer microenvironment is just beginning to emerge. They exhibit both pro- and anti-tumorigenic functions depending on the tissue involved ^100– 103^. While tumor-infiltrating NK cells are often associated with a good prognosis, the accumulation of ILC1s that have transdifferentiated from NK cells is correlated with a loss of anti-tumor protection ^104^. Although immunosuppressive functions have been attributed to ILC2s found in tumors through their production of type 2 cytokines and enhancement of myeloid-derived suppressor cell function ^105^, the role of these cells in tumor immunity is still fairly limited ^106^. A recent study demonstrated that ILC2s could induce potent anti-tumor responses in pancreatic cancer and were associated with anti-PD-1 therapy efficacy ^107^. This elegant work revealed that ILC2s can accumulate in pancreatic tumors and are associated with positive outcomes for patients. IL-33-dependent ILC2 tumor infiltration drove intra-tumoral dendritic cell accumulation, collectively improving anti-tumor immunity. However, IL-33-activated tumor-infiltrating ILC2s expressed PD-1. This normally inhibits their anti-tumor functions but ablation of PD-1 expression, or blocking the interaction with its ligand using monoclonal antibodies, negated this effect and enhanced the anti-tumor response ^107^. ****

Local tissue immunosurveillance is of extreme importance to constrain early tumor development. Increased understanding of tissue-resident cells would allow the design of specific anti-tumor therapeutics for tumor eradication and long-term protection. The dynamic and temporal regulation of circulating and resident lymphocytes has opened new models to help us envisage how immune cells communicate with epithelia, neurons and stromal cells in tissues and coordinate regional and systemic remodelling in response to local perturbations.

## Conclusions

Tissue-resident cells are highly abundant throughout the body. They have been generally considered to be a static population. More recent evidence makes it clear that these cells can sense changes in the environment and implement new programs that allow these cells to move, either locally, or even enter the circulatory system to re-join trafficking immune cells, at least for a short while. At present, we have only a superficial understanding of these regulatory mechanisms. Although being tethered within a tissue offers a strong capacity to effect immunosurveillance, how we can target them therapeutically and deliver precise signals to optimize the positive effector activity of resident immune cells is not clear. Understanding the rules around this pathway offers significant opportunity for vaccine delivery and amplification of engineered cells (for example, chimeric antigen receptor T cells) to target particular tissues.

Key questions remain:

- What does heterogeneity of tissue-resident cells indicate—do they have enhanced or diminished protective functions?
- How can short-lived resident memory cells be modulated to enhance their long-term value for enhancement of barrier protection and thus potential for vaccination or anti-tumor responses at these surfaces?
- While tissue-resident cells are mainly thought to protect barriers, do they impede vaccine or anti-tumor targeting?
- How do we dampen down exuberant tissue-resident responses to prevent disease?
- How are tissue-resident cell numbers regulated? Is this niche-dependent and can tissue-resident cells be amplified to fill niches, or is the number of tissue-resident cells finite and defined homeostatically?

Although the categorization of immune cells as tissue-resident has only recently occurred, this understanding of a dedicated population with properties specific to barrier protection potentially opens many doors to therapeutic targeting and a reassessment of our approaches and previous failures. Future research will undoubtedly uncover new and tangible approaches that might be readily implemented and have immediate impact on treatment and prevention of disease.
